# Linguistic shifts in dental scientific writing in the large language model era

**DOI:** 10.1186/s41073-026-00226-x

**Published:** 2026-06-04

**Authors:** Hadar Better, Doron Haim, Thabet Asbi, Gavriel Chaushu, Keren Better

**Affiliations:** 1 Maccabi-Dent Medical & Quality Assurance Department, Tel-Aviv, 6801298 Israel; 2 Department of Health Systems Management, Faculty of Health Sciences, Ariel University, Ariel, Israel; 3 Maccabi-Dent Research and Innovation Department, Tel-Aviv, 6801298 Israel; 4 Department of Periodontology, Rambam Health Care Campus, Haifa, 3109601 Israel; 5 Department of Oral and Maxillofacial Surgery, Rabin Medical Center, Petah Tiqwa, Israel; 6 Department of Information and Computing Sciences, Utrecht University, Princetonplein 5, Utrecht, 3584 CC The Netherlands

**Keywords:** Research integrity, Artificial intelligence, Large language models, ChatGPT, Scientific writing, Authorship transparency, Publication ethics

## Abstract

**Background:**

The integrity of scientific literature depends on transparent authorship and authentic communication of research findings. The release of ChatGPT in November 2022 introduced powerful AI writing assistance that may be changing how dental researchers write manuscripts. However, systematic evidence documenting this shift and its implications for research integrity remains limited. Traditional detection tools and self-reported surveys have proven unreliable, requiring alternative approaches to assess AI’s influence on published literature.

**Objective:**

To detect and quantify linguistic changes in dental scientific abstracts by comparing pre-ChatGPT (2021) and post-ChatGPT (2025) periods, providing empirical evidence of AI adoption patterns and their implications for scientific communication integrity.

**Methods:**

We analyzed 2,770 abstracts from six major dental journals using vocabulary-based linguistic analysis. Following computational linguistics methods validated in biomedical literature, we extracted six AI-associated features and computed composite detection scores. This observational text-based approach avoids reporting bias while providing quantifiable evidence of linguistic shifts.

**Results:**

AI detection scores increased significantly across all journals (mean: 12.01 to 14.46; + 20.4%), with individual journal increases ranging from +10.0% to +34.5%. The mean number of AI marker words per abstract rose from 0.69 to 0.97 (+41.1%), while high-suspicion abstracts (score ≥ 20) increased from 18.0% to 25.8% (+43%). Changes were consistent across journal specialty, geography, and impact factor, suggesting field-wide adoption.

**Conclusions:**

Dental scientific writing has undergone measurable linguistic changes coinciding with widespread AI availability. The consistency and magnitude of these changes raise questions about transparency in AI use, maintaining authorial voice, and preserving the integrity of scientific communication. These findings provide an empirical foundation for evidence-based policies on AI disclosure and appropriate use in academic publishing, grounded in observed changes to scientific communication rather than speculative concern.

## Introduction

In late 2022, a shift began in scientific writing. Researchers across fields noticed changes in how papers read subtle alterations in word choice, sentence structure, and stylistic patterns. The launch of ChatGPT in November 2022 coincided with these observations, and within months, evidence emerged that large language models were beginning to influence academic writing [1].

In biomedicine, researchers have already documented this shift. Liang and colleagues found that certain words (comprehensive, crucial, complex, meticulous) surged by 50% or more in abstracts published after ChatGPT's release [1]. They used computational linguistics methods [2, 3] to track these changes across nearly a million papers. Their work revealed a clear signature: Large Language Models leave linguistic fingerprints in the text they generate or help refine [1].

But what about dentistry? This field has its own culture, its own journals, its own writing traditions. Would we see the same patterns? Would the changes be universal across specialties, from endodontics to periodontics, from clinical research to basic science? These weren't just academic questions. They touched on issues of scientific integrity, authorship transparency, and the future of how dental researchers communicate their findings. As the findings reported below show, measurable stylometric changes were observed across all six journals examined, regardless of specialty or geographic scope.

As large language models became widely adopted, their impact on scientific writing progressed far more quickly than the policies intended to guide their use. Several biomedical fields have already begun examining how these tools may be shaping the language of published research, yet comparable evidence within dental literature is still lacking.

Although this study focuses on dental journals, dentistry does not represent a methodological or editorial outlier within biomedical research. The structure of scientific articles, peer-review standards, authorship norms, and linguistic conventions in dental research closely mirror those of the broader medical literature. Accordingly, dentistry serves here as a well-defined and internally diverse analytical lens through which broader shifts in scientific writing practices can be examined. Because language is the primary vehicle through which scientific reasoning, accountability, and authorship are communicated, systematic shifts in scientific writing style are not merely linguistic phenomena, but integrity-relevant signals that warrant empirical scrutiny. The patterns observed are therefore not interpreted as discipline-specific anomalies, but as indicative of wider changes in biomedical scientific communication in the era of large language models. While our analysis focuses on dentistry, the integrity implications extend to any field where AI tools are reshaping how scientists write.

Therefore, the aim of this study was to examine whether measurable linguistic changes have occurred in dental journal abstracts over time, and to assess whether any emerging patterns align with the growing influence of large language models on the way dental research is written. It is important to note at the outset that the method employed documents population-level stylometric patterns; it cannot establish AI use in specific manuscripts or attribute individual abstracts to any particular cause.

## Methods

### Study design

We conducted a retrospective bibliometric analysis comparing linguistic patterns in dental journal abstracts published before and after the widespread availability of ChatGPT. We selected January-June 2021 as our pre-AI baseline (18 months before ChatGPT's November 2022 release) and January-June 2025 as our post-AI comparison period (approximately 2.5 years after ChatGPT's release, allowing time for adoption).

### Journal selection

We identified six major dental journals representing diverse specialties, geographic scopes, and impact factors:

- 1. Journal of Dental Research (general, highest impact factor).
- 2. Journal of Endodontics (specialty: endodontics).
- 3. American Journal of Orthodontics and Dentofacial Orthopedics (specialty: orthodontics).
- 4. Clinical Oral Investigations (clinical research, European).
- 5. International Journal of Paediatric Dentistry (specialty: paediatric dentistry).
- 6. Journal of Oral and Maxillofacial Surgery (specialty: oral surgery).

These journals span the spectrum of dental research from basic science to clinical practice, from North American to European editorial boards, and from generalist to specialist audiences.

### Data collection

We downloaded all article abstracts published in these six journals during the two time periods using PubMed’s E-utilities API. We included only original research articles, excluding editorials, letters, case reports, and reviews. Review articles were excluded for two reasons: first, they employ a different linguistic register by design, in which words such as ‘comprehensive’ and ‘critical’ are genre-appropriate and would confound detection of LLM-associated drift in original research; second, restricting the corpus to original research articles provides a more homogeneous basis for longitudinal comparison, since the proportion of reviews relative to original articles may itself have changed between 2021 and 2025. This exclusion means that our estimates are likely conservative: had review articles been included, the magnitude of stylometric drift across all dental literature would probably be larger. For each abstract, we extracted: publication date, journal name, and complete abstract text.

### AI detection methodology

We employed vocabulary-based detection rather than commercial AI detection software. Research has demonstrated that commercial detectors produce unreliable results with high false-positive rates, particularly for academic writing [4, 5]. Instead, we followed methods from computational linguistics that focus on specific linguistic markers shown to increase dramatically after LLM deployment [1–3, 6].

We extracted six features from each abstract, based on established LLM fingerprints identified in biomedical literature [1]:

- 1. AI Marker Words: Frequency of 15 high-weight words shown to surge after ChatGPT (comprehensive, crucial, complex, meticulous, meticulously, handling, complexities, realm, showcasing, tailored, towards, underpins, important, facilitating, insights).
- 2. Excess Adjectives: Proportion of adjectives relative to total words.
- 3. Long Sentences: Proportion of sentences exceeding 30 words.
- 4. Rare Words: Frequency of words outside the 10,000 most common English words.
- 5. Readability: Flesch Reading Ease score (0–100 scale, lower = harder).
- 6. Perplexity Proxy: Vocabulary diversity measured as type-token ratio.

We standardized each feature to z-scores and combined them into a composite AI Suspicion Score using equal weighting. This approach was selected for three reasons: (1) Equal weighting avoids arbitrary decisions about feature importance when validated weights are unavailable, (2) all six features have been independently associated with AI-generated text in prior research [1–3], and (3) equal weighting facilitates replication by other researchers. Higher scores indicate greater likelihood of AI involvement in writing. The composite score is not intended as a classifier of AI authorship, but as a continuous indicator of population-level linguistic drift associated with known large language model fingerprints.

It is important to distinguish this approach from the mixture model framework of Liang et al. [1], who estimated the population proportion of LLM-influenced papers using a trained probabilistic model calibrated against ground-truth human and LLM texts. Our study does not implement a trained classifier or require a labeled reference corpus. Instead, it documents whether stylometric patterns associated with LLM use have shifted over time within a defined corpus. These are complementary approaches operating at different methodological levels. We cite Liang et al. as the source of our AI marker word feature set and as a point of directional comparison, not as methodological validation. The 15 marker words used in this study were adopted directly from Liang et al. [1] without modification; they were identified through an empirical analysis of approximately one million biomedical abstracts and were not selected post-hoc based on our own data. This approach has since been independently supported by large-scale excess vocabulary analyses in biomedical literature.

### Statistical analysis

We compared 2021 with 2025 by reporting mean differences with 95% confidence intervals. We calculated Cohen's d effect sizes to quantify the magnitude of differences, with d = 0.2 considered small, d = 0.5 medium, and d = 0.8 large [7, 8]. We analyzed data both across all journals combined and separately for each journal to assess consistency of findings. Because abstracts are subject to editorial standardization, any observed increases are more likely to underestimate than exaggerate underlying changes in writing practices. We also examined the proportion of abstracts exceeding a high-suspicion threshold (score ≥ 20, representing approximately the top 18% of all abstracts in the pre-AI baseline period). To account for multiple comparisons across the six journals, Benjamini–Hochberg false discovery rate (FDR) correction was applied to the journal-level p-values. FDR-corrected values are reported in Table 1.

**Table 1:** AI detection scores by journal and year

Journal	n (2021)	n (2025)	2021 Mean (SD)	2025 Mean (SD)	Change	*p*-value (FDR-adj.)
JOE	191	148	12.04 (6.98)	16.20 (9.73)	+ 34.5%	< 0.001
ORTHO	245	130	9.40 (8.72)	11.90 (9.81)	+ 26.6%	0.012
IJPD	90	103	9.76 (5.40)	12.20 (7.24)	+ 25.0%	0.009
JOMS	411	159	9.85 (7.60)	11.96 (7.91)	+ 21.4%	0.003
JDR	170	146	14.61 (10.46)	17.24 (10.57)	+ 18.0%	0.028
COI	648	329	13.98 (8.17)	15.38 (9.68)	+ 10.0%	0.018
TOTAL	1,755	1,015	12.01 (8.39)	14.46 (9.56)	+ 20.4%	< 0.001

Mean AI detection scores are presented as mean (standard deviation [SD]). Change values represent relative increase from 2021 to 2025

*p*-values are adjusted for multiple comparisons across the six journals using the Benjamini–Hochberg false discovery rate correction

### Ethical considerations

This study analyzed publicly available published abstracts and involved no human participants. No individual authors are identified in our analysis. We report aggregate patterns across journals rather than flagging specific articles or authors.

### AI use statement

This manuscript was written by the authors. Claude (Anthropic) was used to assist with Python scripting for data processing in Google Colab, including text parsing, feature computation, and data organization. No part of the manuscript text was written, edited, or revised using AI tools. All scientific content, interpretations, conclusions, and writing decisions were made independently by the authors.

## Results

**Fig. 1:**
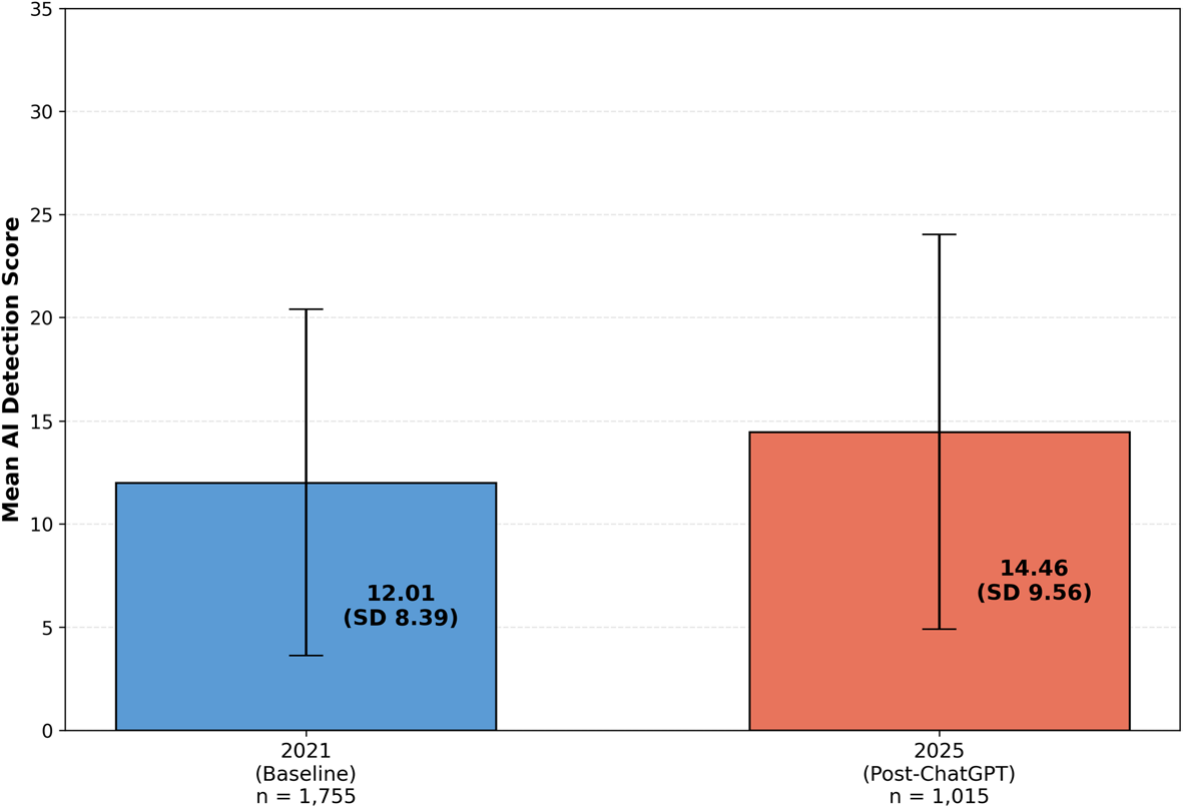
Mean AI detection scores in dental journal abstracts, 2021 versus 2025. Bars show mean composite AI suspicion scores; error bars represent standard deviation. n = 1,755 (2021) and n = 1,015 (2025). Mean difference 2.45 (95% CI 1.77 to 3.13); Cohen d = 0.27

**Fig. 2:**
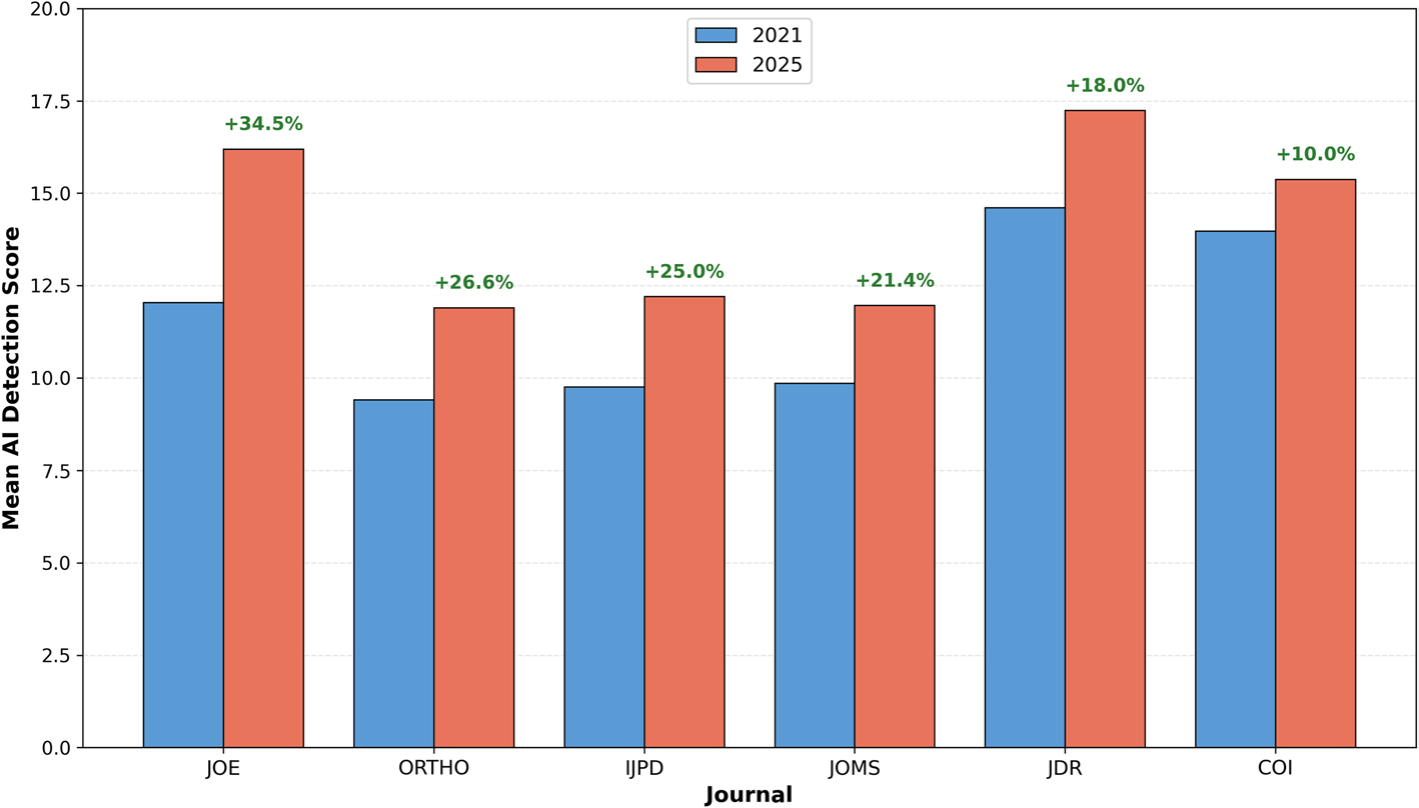
AI detection scores by journal, 2021 versus 2025. Bars show mean composite AI suspicion scores per journal. Percentage values indicate relative change between time periods. JOE = Journal of Endodontics; ORTHO = American Journal of Orthodontics and Dentofacial Orthopedics; IJPD = International Journal of Paediatric Dentistry; JOMS = Journal of Oral and Maxillofacial Surgery; JDR = Journal of Dental Research; COI = Clinical Oral Investigations

AI detection scores increased significantly from 2021 to 2025 (Fig. 1). The mean score rose from 12.01 (standard deviation [SD] 8.39) in 2021 to 14.46 (SD 9.56) in 2025, a mean difference of 2.45 (95% CI 1.77 to 3.13), corresponding to a 20.4% relative increase (Cohen d = 0.27). This change was consistent across all six journals analyzed (Fig. 2).

Individual journal increases ranged from 10.0% to 34.5% (Table 1). The Journal of Endodontics showed the largest absolute increase (+4.16 points), as well as the largest relative increase (+34.5%), while Clinical Oral Investigations showed the smallest relative increase (+10.0%). All changes were statistically significant after Benjamini–Hochberg false discovery rate correction (Table 1), with small to medium effect sizes (d = 0.16 to 0.50).

Abstract length differed modestly between periods (2021 mean 214.3 words [SD 96.7]; 2025 mean 231.2 words [SD 94.6]; mean difference 16.9 words, 95% CI 9.5 to 24.3). To assess whether this length difference could explain the observed AI score changes, we examined the correlation between per-journal length change and per-journal AI score change. This correlation was r = 0.26 (*p* = 0.62), near zero and not statistically significant. Three journal-level examples illustrate the dissociation: Clinical Oral Investigations showed a length decrease of 5 words yet recorded a + 10.0% AI score increase; the Journal of Oral and Maxillofacial Surgery showed the largest length increase (+56 words) but only a + 21.4% AI score increase; the International Journal of Paediatric Dentistry showed virtually no length change (+3.6 words) yet a + 25.0% AI score increase. These patterns indicate that abstract length does not account for the observed stylometric drift.

An internal consistency analysis further supports the coherence of the composite index. Abstracts containing at least one AI marker word scored a mean of 20.59 (SD 7.99), compared to 6.68 (SD 2.39) for abstracts without any marker words. All abstracts scoring ≥ 15 on the composite index contained at least one marker word (100%). The correlation between marker word count and composite score was r = 0.978. These findings indicate that high composite scores are not produced by the remaining six features independently of marker word presence, and that the index behaves with strong internal consistency.

The patterns varied meaningfully across journals. The Journal of Endodontics demonstrated the most pronounced shift, with abstracts from 2025 showing 34.5% higher AI suspicion scores than those from 2021. In contrast, Clinical Oral Investigations and the Journal of Oral and Maxillofacial Surgery showed smaller increases, with only 10–21% changes. The Journal of Dental Research, American Journal of Orthodontics and Dentofacial Orthopedics, and International Journal of Paediatric Dentistry fell between these extremes, each showing increases of approximately 20%.

**Fig. 3:**
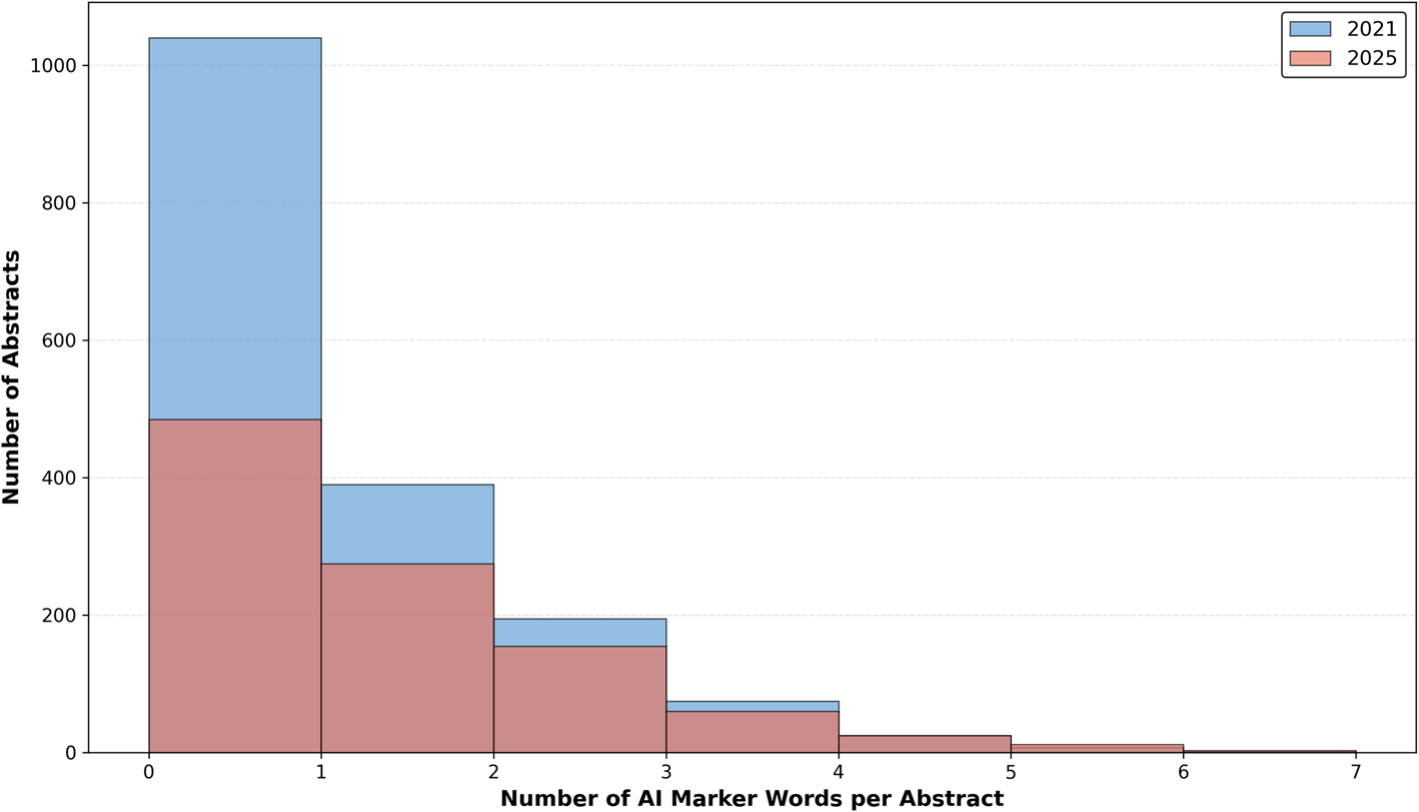
Distribution of AI marker word frequency per abstract, 2021 versus 2025. Histograms show the number of abstracts containing each count of AI marker words (from the predefined 15-word list). 2021 mean = 0.69; 2025 mean = 0.97 (relative change +41.1%)

AI marker word usage increased markedly (Fig. 3). The mean number of AI marker words per abstract rose from 0.69 in 2021 to 0.97 in 2025, a 41.1% increase. The most common markers in 2025 were 'comprehensive' (5.1% of abstracts), 'crucial' (4.1%), and 'insights' (3.8%). Several words that were essentially absent in 2021 became measurable in 2025, including 'meticulously, ' 'showcasing, ' and 'facilitating [1].'.

These shifts in vocabulary were not subtle. The word 'insights' appeared 6 times more frequently in 2025 abstracts (3.8%) compared to 2021 (0.6%), while 'crucial' showed a 4-fold increase (1.0% to 4.1%). Words like 'highlight' and 'complex'—each appearing in fewer than 0.2% of 2021 abstracts—became 5–7 times more common by 2025. The pattern extended beyond individual words to entire phrases. The template construction "this study aimed to" appeared in 7.4% of 2021 abstracts but 15.6% of 2025 abstracts, more than doubling in just four years.

**Fig. 4:**
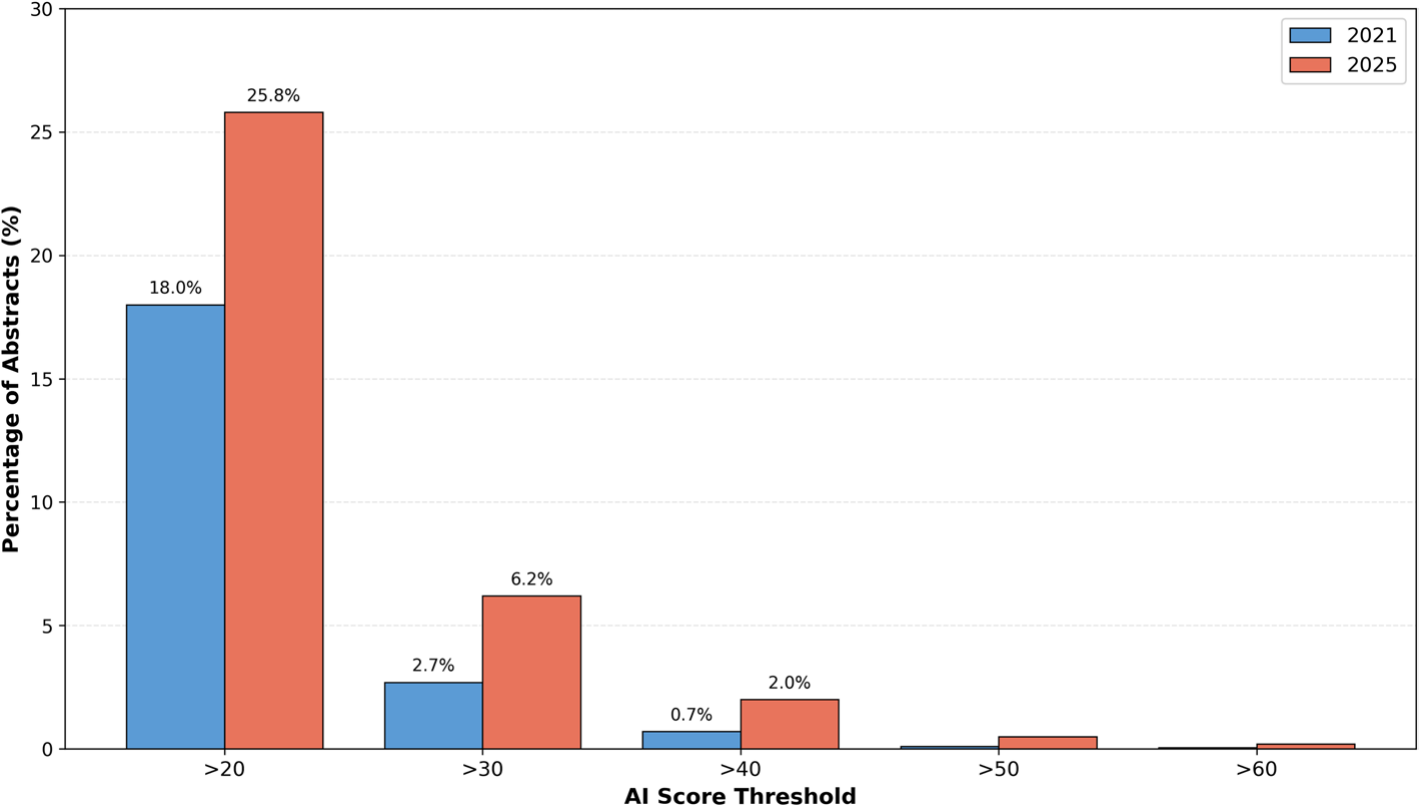
Prevalence of high AI detection scores in dental journal abstracts, 2021 versus 2025. Bars show the percentage of abstracts exceeding each composite AI suspicion score threshold (> 20, > 30, > 40, > 50, > 60)

The proportion of high-suspicion abstracts (score ≥ 20) increased from 18.0% in 2021 to 25.8% in 2025, a 43% relative increase (Fig. 4). This finding indicates that not only did average scores increase, but a substantial proportion of abstracts showed particularly strong stylometric drift.

The increases were remarkably consistent regardless of journal characteristics. Journals with higher impact factors showed similar patterns to those with lower impact factors. Specialty journals (e.g., Journal of Endodontics, American Journal of Orthodontics and Dentofacial Orthopedics) showed similar patterns to general journals (e.g., Journal of Dental Research). European journals (e.g., Clinical Oral Investigations) showed similar patterns to North American journals. This consistency suggests a field-wide phenomenon rather than isolated adoption by specific research communities.

## Discussion

### Principal findings

This study provides systematic evidence of measurable stylometric changes in dental scientific abstracts between 2021 and 2025. Across 2,770 abstracts from six major journals, we observed a 20.4% increase in composite linguistic drift scores and a 41.1% increase in AI marker word usage. These changes were consistent across journals regardless of specialty, geography, or impact factor. The temporal and directional pattern is consistent with the growing availability and adoption of LLM writing tools, though the data document linguistic change and cannot establish its cause at the individual author level.

Our findings align with patterns documented in broader biomedical literature. 1 Liang and colleagues reported increases of 50–100% in AI marker words across bioRxiv and PubMed abstracts after ChatGPT's release [1]. While our 41.1% increase in AI marker word usage is somewhat smaller, it follows the same direction and suggests that dental research is participating in the same transformation affecting scientific writing generally.

### Interpretation and implications

The between-journal variation in the magnitude of change warrants comment. The Journal of Endodontics showed the most pronounced shift (+34.5%), which may reflect that endodontic research articles, focused on procedural and technical topics, are particularly amenable to LLM-assisted elaboration when authors seek to improve clarity. In contrast, the Journal of Oral and Maxillofacial Surgery showed a smaller increase (+21.4%), which may reflect the procedure-focused, technical nature of surgical writing, leaving less room for the elaborate descriptive language that characterizes LLM output. These are interpretive observations based on specialty characteristics and should be treated as hypotheses rather than established findings.

These findings raise questions about scientific publishing in the AI era. Major journals have responded with varying policies. Science has taken a firm stance, stating that text generated by AI tools constitutes plagiarism and is prohibited [9]. Nature requires authors to disclose AI use and prohibits listing AI tools as authors [10]. JAMA discourages AI-generated text but requires citation when used [11]. These different approaches reflect uncertainty about how to govern AI in academic writing. The AI tools available during our January 2025 data collection period were considerably more sophisticated than those at ChatGPT's November 2022 release. Subsequent models like GPT-4 (released March 2023) and Claude 3 (March 2024) generate increasingly sophisticated, human-like text with better contextual awareness and stylistic flexibility. The linguistic shifts we document therefore reflect not merely AI adoption, but adoption of progressively advanced tools. This temporal dimension has significant implications: as AI continues to evolve toward more human-like output, vocabulary-based detection, while more reliable than commercial tools, may also face growing challenges. The stylometric patterns we detect may therefore already be less pronounced than those produced by earlier, more formulaic LLM outputs, and the gap between human and AI writing styles may continue to narrow. This has a direct bearing on policy: if technical detection is inherently reactive and will always lag behind model development, then transparent author disclosure becomes the more durable solution for maintaining research integrity. This evolutionary trajectory underscores why policy-based transparency requirements are essential complements to technical detection methods.

The consistency of changes across our six journals suggests that AI adoption in dental writing is widespread and cuts across different research communities. This raises concerns about a potential homogenization of scientific writing-if similar AI tools are widely adopted, stylistic variance across authors and journals may gradually diminish, potentially affecting the expression of individual scholarly voice. Or will AI simply serve as a more sophisticated grammar checker, helping non-native English speakers compete on equal footing with native speakers? Of note, the three Elsevier journals in our sample had no explicit AI-related guidance at the time of access, while only Clinical Oral Investigations had publisher-level guidance during our data collection period.

Some argue AI offers clear benefits: increased efficiency, improved clarity, assistance for non-native English speakers, and democratization of high-quality scientific writing [12, 13]. Others worry about risks: loss of authorial voice, potential for introducing subtle errors that sound plausible [14], reduced development of writing skills among trainees, and questions about intellectual contribution and accountability [13, 15].

Our study cannot resolve these debates, but it provides empirical grounding. The changes are real, measurable, and substantial. Whatever policies the dental research community adopts, they should be based on accurate understanding of how extensively AI is already being used.

### Policy implementation and timing

The linguistic shifts documented in our 2025 sample occurred during a period of evolving AI disclosure policies in dental publishing. During our study periods (2021 and January 2025), formal AI disclosure requirements were either absent or newly emerging across the journals examined. Publisher-level AI policies began appearing in 2024 (Elsevier, Springer Nature, Wiley), but journal-specific implementation varied considerably. For example, the Journal of Dental Research published explicit AI guidance only in October 2025, 18 months after our data collection period.

This temporal gap between observable AI adoption, as reflected in linguistic patterns observed in abstracts published in January 2025, and the availability of explicit, journal-level disclosure guidance represents a substantive challenge for research integrity. During the study period, the absence of mandatory or consistently articulated disclosure frameworks means that AI-assisted writing, where present, may not have been systematically disclosed. This concern is reinforced by recent evidence that AI-generated content has appeared in peer-reviewed journals indexed in major scientific databases without author declaration, including in high-impact journals across medicine, engineering, and computer science [16]. This policy lag underscores the importance of developing standardized, field-specific disclosure approaches that align more closely with evolving writing practices, a convergence that, based on our findings, appears to have occurred later than the onset of widespread AI adoption in dental scientific writing (Table 2). Table 2 summarizes the availability of AI-related guidance across all six journals for both study periods (2021 baseline and 2025 sample).

**Table 2:** Availability of AI-related guidance across six dental journals

Journal	Publisher	2021 Baseline Period	2025 Sample period (January–June 2025)
Journal of Dental Research	SAGE	No journal-specific AI guidance	No journal-specific AI guidance*
Journal of Endodontics	Elsevier	No journal-specific AI guidance	No journal-specific AI guidance
American Journal of Orthodontics and Dentofacial Orthopedics	Elsevier	No journal-specific AI guidance	No journal-specific AI guidance
Clinical Oral Investigations	Springer Nature	No journal-specific AI guidance	Publisher-level guidance (2024)
International Journal of Paediatric Dentistry	Wiley	No journal-specific AI guidance	No journal-specific AI guidance*
Journal of Oral and Maxillofacial Surgery	Elsevier	No journal-specific AI guidance	No journal-specific AI guidance

^*^ Journal-specific guidance published after the 2025 sample period

All journals lacked journal-specific AI disclosure guidance during the 2021 baseline period. During the 2025 sample period (January–June 2025), only Clinical Oral Investigations had publisher-level AI guidance available (implemented in 2024). Three journals (Journal of Endodontics, American Journal of Orthodontics and Dentofacial Orthopedics, Journal of Oral and Maxillofacial Surgery) continued to lack journal-specific guidance. Two journals (Journal of Dental Research, International Journal of Paediatric Dentistry) published journal-specific guidance after the 2025 sample period. Journal policies were reviewed in November 2025. This timeline indicates that the linguistic shifts documented in both study periods occurred in the absence of journal-specific AI disclosure requirements

### Methodological considerations

We used vocabulary-based detection rather than commercial AI detection tools because research consistently shows these tools perform poorly on academic writing [4, 5]. They produce high false-positive rates and can incorrectly flag human written text, particularly from non-native English speakers. Our approach focused on linguistic features that have been empirically validated as distinguishing AI-assisted writing from purely human writing.

That said, we acknowledge several limitations. Our detection method measures statistical associations, not definitive AI use. Some abstracts scoring high on our scale might be purely human written by authors who favor formal, detailed prose. Conversely, some abstracts with low scores might have received AI assistance in ways our features don't capture (e.g., help with study design rather than writing). We can detect patterns consistent with AI involvement but cannot prove specific instances of AI use [1]. The composite index has not been validated against a ground-truth labeled corpus of AI-assisted and non-AI-assisted texts; the internal consistency evidence reported above supports construct coherence but does not constitute external validation. Before this index can be used to make claims about AI involvement in individual texts, it must first be validated against a labeled reference corpus that includes human-authored, AI-generated, AI-edited, and professionally language-edited abstracts. We did not perform such validation here. It is the necessary next step. Several additional confounding factors cannot be ruled out: (1) AI-assisted language editing services grew substantially between 2021 and 2025, meaning authors who did not use LLMs directly may still have used AI-powered style tools that produce similar lexical patterns; (2) some journals updated their structured abstract requirements during this period, potentially influencing vocabulary in ways that overlap with LLM-associated patterns; (3) exposure to a growing body of AI-assisted literature may have gradually influenced authors' own writing styles independent of direct tool use; and (4) shifts in the demographic composition of submitting authors may contribute, as non-native English speakers who adopt formal vocabulary may affect the observed drift regardless of AI tool use. These factors mean that our findings should be interpreted as documenting linguistic change rather than establishing its cause.

We analyzed only abstracts, which represent the most visible and carefully edited portion of papers but constitute a small fraction of total text. Full-text analysis might reveal different patterns. We also focused on a limited time window and six journals-broader sampling across more journals and longer time periods would strengthen conclusions. A key consideration for any detection methodology is the evolving relationship between detection and evasion. As detection methods improve, AI models are simultaneously being refined to produce more human-like text, creating an ongoing 'arms race' between detection capabilities and AI development. Vocabulary based approaches like ours are less susceptible to targeted evasion than signature-based commercial detectors, since they rely on statistical patterns across multiple linguistic features rather than specific markers that can be deliberately avoided. However, future AI iterations designed specifically to minimize linguistic fingerprints could reduce detection efficacy over time. This changing context reinforces our argument that policy-based solutions-mandatory disclosure and transparent authorship guidelines must take precedence over purely technical detection approaches, which may always lag behind AI capabilities.

### Implications for policy and practice

Our findings suggest that dental journals should clarify their AI policies and implement clear disclosure requirements that specify when and how AI use should be reported [17]. The data demonstrate that delaying policy implementation until after widespread AI adoption-as occurred in dental literature between 2024–2025 may result in a substantial body of undisclosed AI-assisted work in the published record.

At minimum, transparency seems essential. Authors should disclose AI use in a dedicated statement within the manuscript, similar to declarations for statistical support or medical writing services. This disclosure should specify which manuscript sections received AI assistance (e.g., “AI tools were used to improve clarity of the discussion section” vs. “AI tools were not used in manuscript preparation”). But disclosure alone may be insufficient. Journals should consider establishing clear boundaries: AI assistance for language editing and formatting may be acceptable, while AI generation of scientific reasoning, data interpretation, or literature synthesis may require additional scrutiny or may be prohibited [15].

Reviewers and editors should also adapt. If AI-assisted writing is becoming standard, reviewers might need training to identify when AI is being used inappropriately (e.g., to generate fabricated citations or mask poor-quality research). Editors might need new tools or workflows to detect problematic AI use while not penalizing appropriate assistance.

Future research should examine whether AI use correlates with other publication characteristics like citation rates, retractions, or quality indicators. Longitudinal studies tracking individual authors' linguistic patterns before and after AI adoption would provide stronger evidence than our cross-sectional design. Qualitative research interviewing dental researchers about their AI use would complement our quantitative findings and help understand motivations, perceived benefits, and concerns. A multi-decade comparison sampling from years such as 2010, 2015, and 2019 alongside 2021 and 2025 would allow researchers to determine whether the observed shift is temporally specific to the post-LLM period or reflects a longer secular trend in scientific writing. This question has been addressed at scale in the broader biomedical literature: Kobak and colleagues analyzed over 15 million PubMed abstracts from 2010 to 2024 and found that vocabulary changes after 2022 were without precedent in any prior year, including during the COVID-19 pandemic period, supporting the temporal specificity of LLM-associated stylometric drift [6]. The same approach applied to dental-specific corpora over a longer time horizon would strengthen the conclusions of the present study.

## Conclusion

Dental scientific abstracts show measurable stylometric changes between 2021 and 2025 that are directionally consistent with LLM-associated linguistic patterns documented in the broader biomedical literature. The changes are statistically significant, consistent across journals of different types and publishers, and of a magnitude that warrants attention from the research community. What drove these changes cannot be established from these data alone, but the temporal and directional pattern supports their relevance to ongoing discussions about AI use, authorship transparency, and disclosure policy in academic publishing. As AI becomes increasingly integrated into the research workflow, the dental research community must engage thoughtfully with questions of transparency, authorship, and maintaining the integrity of scientific communication.

## Data Availability

All data underlying the findings of this study, including numerical values used for statistical analyses and figure generation, are publicly available at (10.5281/zenodo.17920369).
